# (1*R*,3a*R*,5a*S*,6*S*,8a*R*,8b*R*,9a*S*)-1-Hydr­oxy-6-isopropyl-1,3a,5a-trimethyl­perhydro­cyclo­penta­[*a*]cyclo­propa[*i*]naphthalen-4-one

**DOI:** 10.1107/S1600536810001169

**Published:** 2010-01-16

**Authors:** Iván Brito, Jorge Bórquez, Luis Alberto Loyola, Michael Bolte, Joselyn Albanez

**Affiliations:** a Departamento de Química, Facultad de Ciencias Básicas, Universidad de Antofagasta, Casilla 170, Antofagasta, Chile; b Institut für Anorganische Chemie der Goethe-Universität Frankfurt, Max-von-Laue-Strasse 7, D-60438 Frankfurt am Main, Germany

## Abstract

The title compound (also know as azorellanone), C_20_H_32_O_2_, is built up from three fused carbocycles, one five-membered ring and two six-membered rings. The five membered-ring has an envelope conformation, whereas the six-membered rings have a distorted half-chair and a twist–boat conformation. In the crystal, mol­ecules are linked by O—H⋯O inter­actions into zigzag chains with graph-set notation *C*(8) along [010]. The absolute configuration was assigned on the basis of earlier chemical studies.

## Related literature

For related structures, see: Loyola *et al.* (1998 ▶, 2000 ▶, 2001 ▶, 2004 ▶); Borquez *et al.* (2007 ▶). For the biological properties of diterpenoids with azorellane and mulinane skeletons, see: Chiaramello *et al.* (2003 ▶); Fuentes *et al.* (2005 ▶); Delporte *et al.* (2003 ▶); Morales *et al.* (2003 ▶); Neira *et al.* (1998 ▶). For graph-set notation, see: Bernstein *et al.* (1995 ▶). For a description of the Cambridge Structural Database, see: Allen (2002 ▶). For puckering parameters, see: Cremer & Pople (1975 ▶).
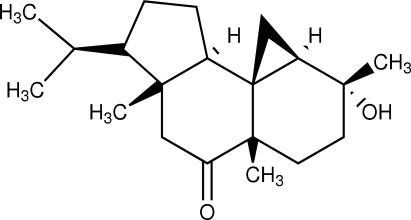


## Experimental

### 

#### Crystal data

C_20_H_32_O_2_
*M* *_r_* = 304.46
Monoclinic, 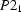

*a* = 6.0073 (5) Å
*b* = 13.3348 (11) Å
*c* = 11.2743 (8) Å
β = 99.271 (6)°
*V* = 891.34 (12) Å^3^
*Z* = 2
Mo *K*α radiation
μ = 0.07 mm^−1^
*T* = 173 K
0.37 × 0.36 × 0.36 mm

#### Data collection

Stoe IPDSII two-circle diffractometer
6336 measured reflections
2107 independent reflections
1876 reflections with *I* > 2σ(*I*)
*R*_int_ = 0.066

#### Refinement

*R*[*F* ^2^ > 2σ(*F* ^2^)] = 0.041
*wR*(*F* ^2^) = 0.100
*S* = 1.00
2107 reflections
204 parameters
1 restraint
H atoms treated by a mixture of independent and constrained refinement
Δρ_max_ = 0.2 e Å^−3^
Δρ_min_ = −0.16 e Å^−3^

### 

Data collection: *X-AREA* (Stoe & Cie, 2001 ▶); cell refinement: *X-AREA*; data reduction: *X-AREA*; program(s) used to solve structure: *SHELXS97* (Sheldrick, 2008 ▶); program(s) used to refine structure: *SHELXL97* (Sheldrick, 2008 ▶); molecular graphics: *XP* in *SHELXTL-Plus* (Sheldrick, 2008 ▶); software used to prepare material for publication: *SHELXL97*.

## Supplementary Material

Crystal structure: contains datablocks global, I. DOI: 10.1107/S1600536810001169/fl2288sup1.cif

Structure factors: contains datablocks I. DOI: 10.1107/S1600536810001169/fl2288Isup2.hkl

Additional supplementary materials:  crystallographic information; 3D view; checkCIF report

## Figures and Tables

**Table 1:** Hydrogen-bond geometry (Å, °)

*D*—H⋯*A*	*D*—H	H⋯*A*	*D*⋯*A*	*D*—H⋯*A*
O1—H1⋯O2^i^	0.99 (3)	1.93 (3)	2.916 (2)	172 (3)

Symmetry code: (i) 
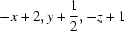
.

**Fig. 1.**
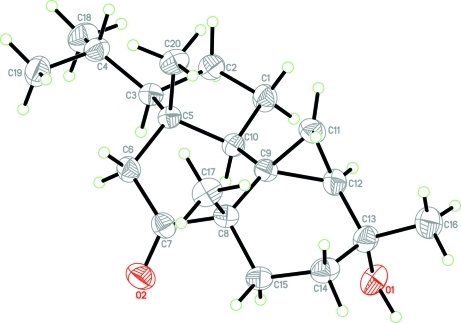
Molecular structure of the title compound with displacement ellipsoids at the 50% probability level showing atom-labelling scheme.

**Fig. 2.**
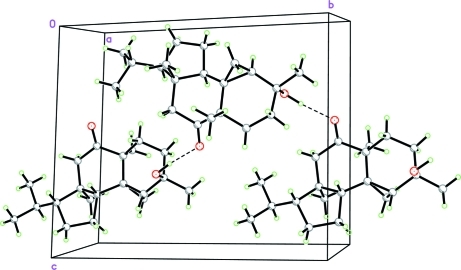
Part of the crystal structure of (I), showing the formation of a C(8) chain along [010]. Hydrogen bond shown as dashed lines.

## supplementary crystallographic information

### Comment

Compounds belonging to the *Azorella, Laretia y Mulinum *genus are recognized as important sources of novel diterpenoids with azorellane and mulinane skeletons (Loyola *et al.*, 1998, 2000; Chiaramello *et al.*, 2003). These metabolites display a wide variety of biological activities, including trichomonicidal, (Loyola *et al.*, 2001), anti-inflammatory and analgesic, (Delporte *et al.*, 2003; Borquez *et al.*, 2007) contraceptive, (Morales *et al.*, 2003) trypanocidal, (Neira *et al.*, 1998) anti-plasmodial (Loyola *et al.*, 2004) and anti-hyperglycemic (Fuentes *et al.*, 2005).

The  title compound (Fig. 1) is built up from three fused carbocycles:  a six membered ring (A) with a methylene bridge between C9 and C12 with a second six membered ring (B) *trans*-fused to a five membered ring (C). The five- membered ring has an envelope conformation whereas the six-membered rings have a distorted half-chair (A) and atwist boat conformation (B) respectively [Q_2_=0.441 (2) Å, φ =112.5 (3)°; Q_T_= 0.518 (2) Å, θ = 48.8 (2)°, φ =272.2 (3)°; Q_T_= 0.677 (2) Å, θ = 97.1 (2)°, φ_2_ = 131.5 (2)°] (Cremer & Pople, 1975). The cyclopropane ring (C9, C11 and C12) features an almost regular triangle with the C9 and C12 distance being slightly longer than the others. The isopropyl, methyl groups at C3, C8, C13 and cyclopropane ring are β-oriented, whereas the hydroxyl group is α-oriented.

A search of the Cambridge Structural Database (CSD, Version 5.31; Allen, 2002) shows no significant variations of the molecular geometry of (I) and the conformations of two closely related compound, azorellanol (CSD refcode FIHYAW; Loyola, *et al.*, 1998) and 7-deacetylazorellanol (CSD refcode NEMXUY; Loyola, *et al.*, 2001).

In the crystal, the molecules are linked by O—H···O interactions into zigzag chains with graph-set notation C(8) along [010] (Bernstein *et al.*, 1995). Atom O1 at (*x*, *y*, *z*) acts as a hydrogen-bond donor to atom O2 at (-*x* + 2,*y* + 1/2,-*z* + 1), (Table1, Fig. 2). The absolute configuration was assigned on the basis of early chemical studies (Loyola *et al.*, 1998).

### Experimental

*Azorella yareta Hauman *plants were collected in Quebradas de las llaretas in Vallenar, Chile. The dried and finely powdered whole plant (1.5 kg) was extracted with petrol ether at room temperature to give a gum (85 g). The concentrated petrol ether extract was fractionated on a silica gel column with hexane-ethyl acetate mixtures of increasing polarity as elution solvents. The fraction (3.45 g) eluted was further separated and purified by silica gel chromatography to give 155 mg of the title compound (also know as azorellanone). Recrystallization from hexane-ethyl acetate (1:1) at room temperature afforded colourless crystals suitable for X-ray diffraction analysis.

### Refinement

In the absence of anomalous scatterers the absolute configuration could not be determined and therefore Friedel pairs were merged. The hydroxyl H atom was refined isotropically. Other H atoms were placed in idealized positions and treated as riding atoms with C—H distances in the range 0.98–1.00 Å and *U*_iso_(H) = 1.2*U*_eq_(C) or 1.5*U*_eq_(C_methyl_).

### Crystal data

**Table:**

C_20_H_32_O_2_	*F*(000) = 336
*M**_r_* = 304.46	*D*_x_ = 1.134 Mg m^−^^3^
Monoclinic, *P*2_1_	Mo *K*α radiation, λ = 0.71073 Å
Hall symbol: P 2yb	Cell parameters from 6216 reflections
*a* = 6.0073 (5) Å	θ = 3.5–27.8°
*b* = 13.3348 (11) Å	µ = 0.07 mm^−^^1^
*c* = 11.2743 (8) Å	*T* = 173 K
β = 99.271 (6)°	Block, colourless
*V* = 891.34 (12) Å^3^	0.37 × 0.36 × 0.36 mm
*Z* = 2	

### Data collection

**Table:**

Stoe IPDSII two-circle diffractometer	*R*_int_ = 0.066
graphite	θ_max_ = 27.5°, θ_min_ = 3.4°
ω scans	*h* = −7→7
6336 measured reflections	*k* = −17→17
2107 independent reflections	*l* = −13→14
1876 reflections with *I* > 2σ(*I*)	

### Refinement

**Table:**

Refinement on *F*^2^	Secondary atom site location: difference Fourier map
Least-squares matrix: full	Hydrogen site location: inferred from neighbouring sites
*R*[*F*^2^ > 2σ(*F*^2^)] = 0.041	H atoms treated by a mixture of independent and constrained refinement
*wR*(*F*^2^) = 0.100	*w* = 1/[σ^2^(*F*_o_^2^) + (0.065*P*)^2^] where *P* = (*F*_o_^2^ + 2*F*_c_^2^)/3
*S* = 1.00	(Δ/σ)_max_ < 0.001
2107 reflections	Δρ_max_ = 0.2 e Å^−^^3^
204 parameters	Δρ_min_ = −0.16 e Å^−^^3^
1 restraint	Extinction correction: *SHELXL97* (Sheldrick, 2008)
Primary atom site location: structure-invariant direct methods	Extinction coefficient: 0.035 (6)

### Special details

**Table:**

Geometry. All e.s.d.'s (except the e.s.d. in the dihedral angle between two l.s. planes) are estimated using the full covariance matrix. The cell e.s.d.'s are taken into account individually in the estimation of e.s.d.'s in distances, angles and torsion angles; correlations between e.s.d.'s in cell parameters are only used when they are defined by crystal symmetry. An approximate (isotropic) treatment of cell e.s.d.'s is used for estimating e.s.d.'s involving l.s. planes.
Refinement. Refinement of *F*^2^ against ALL reflections. The weighted *R*-factor *wR* and goodness of fit *S* are based on *F*^2^, conventional *R*-factors *R* are based on *F*, with *F* set to zero for negative *F*^2^. The threshold expression of *F*^2^ > σ(*F*^2^) is used only for calculating *R*-factors(gt) *etc*. and is not relevant to the choice of reflections for refinement. *R*-factors based on *F*^2^ are statistically about twice as large as those based on *F*, and *R*- factors based on ALL data will be even larger.

### Fractional atomic coordinates and isotropic or equivalent isotropic displacement parameters (Å^2^)

**Table:**

	*x*	*y*	*z*	*U*_iso_*/*U*_eq_	
O1	1.0462 (3)	0.74056 (12)	0.32053 (17)	0.0400 (4)	
H1	1.088 (5)	0.801 (2)	0.369 (3)	0.053 (8)*	
O2	0.8592 (3)	0.42953 (12)	0.55110 (15)	0.0470 (5)	
C1	0.8182 (4)	0.44374 (16)	0.0885 (2)	0.0340 (4)	
H1A	0.9569	0.4797	0.0761	0.041*	
H1B	0.688	0.4727	0.0344	0.041*	
C2	0.8394 (3)	0.33017 (15)	0.06515 (19)	0.0313 (4)	
H2A	0.9912	0.3144	0.0467	0.038*	
H2B	0.7253	0.3089	−0.0036	0.038*	
C3	0.8004 (3)	0.27562 (15)	0.18129 (18)	0.0261 (4)	
H3	0.9506	0.2684	0.2336	0.031*	
C4	0.7041 (3)	0.16952 (15)	0.1557 (2)	0.0311 (4)	
H4	0.5664	0.1751	0.0931	0.037*	
C5	0.6621 (3)	0.35294 (14)	0.24246 (18)	0.0246 (4)	
C6	0.6819 (4)	0.34028 (15)	0.38011 (19)	0.0308 (4)	
H6A	0.5353	0.3164	0.3987	0.037*	
H6B	0.7956	0.2878	0.4068	0.037*	
C7	0.7475 (3)	0.43507 (15)	0.45144 (19)	0.0302 (4)	
C8	0.6549 (3)	0.53588 (15)	0.40016 (18)	0.0271 (4)	
C9	0.6806 (3)	0.54398 (14)	0.26617 (18)	0.0272 (4)	
C10	0.7838 (3)	0.45080 (14)	0.21941 (18)	0.0254 (4)	
H10	0.938	0.4451	0.2679	0.03*	
C11	0.5111 (4)	0.60649 (17)	0.1836 (2)	0.0364 (5)	
H11A	0.4639	0.5823	0.1003	0.044*	
H11B	0.3916	0.6409	0.2192	0.044*	
C12	0.7496 (4)	0.64378 (15)	0.2178 (2)	0.0340 (5)	
H12	0.8433	0.6374	0.1525	0.041*	
C13	0.8031 (4)	0.73557 (15)	0.2965 (2)	0.0356 (5)	
C14	0.7134 (4)	0.72394 (15)	0.4149 (2)	0.0357 (5)	
H14A	0.7752	0.7782	0.4706	0.043*	
H14B	0.5471	0.7305	0.4	0.043*	
C15	0.7781 (3)	0.62273 (15)	0.47304 (19)	0.0307 (4)	
H15A	0.7402	0.6221	0.5553	0.037*	
H15B	0.943	0.613	0.4795	0.037*	
C16	0.7113 (5)	0.83020 (18)	0.2302 (3)	0.0524 (7)	
H16A	0.7472	0.8885	0.2827	0.079*	
H16B	0.5473	0.8245	0.2075	0.079*	
H16C	0.7803	0.8384	0.1577	0.079*	
C17	0.4059 (4)	0.53345 (18)	0.4215 (2)	0.0370 (5)	
H17A	0.3306	0.5959	0.3921	0.055*	
H17B	0.402	0.5264	0.5077	0.055*	
H17C	0.3281	0.4765	0.3782	0.055*	
C18	0.8759 (4)	0.10463 (17)	0.1047 (2)	0.0425 (5)	
H18A	0.9196	0.1379	0.0344	0.064*	
H18B	0.8087	0.0392	0.0809	0.064*	
H18C	1.0095	0.0951	0.1661	0.064*	
C19	0.6366 (5)	0.11760 (17)	0.2651 (2)	0.0429 (5)	
H19A	0.5275	0.1594	0.2985	0.064*	
H19B	0.7706	0.1076	0.3261	0.064*	
H19C	0.5682	0.0525	0.2411	0.064*	
C20	0.4160 (3)	0.35344 (16)	0.1790 (2)	0.0338 (4)	
H20A	0.4119	0.361	0.0922	0.051*	
H20B	0.3356	0.4095	0.2092	0.051*	
H20C	0.3435	0.2902	0.1952	0.051*	

### Atomic displacement parameters (Å^2^)

**Table:**

	*U*^11^	*U*^22^	*U*^33^	*U*^12^	*U*^13^	*U*^23^
O1	0.0403 (8)	0.0284 (7)	0.0521 (10)	−0.0029 (6)	0.0101 (7)	−0.0037 (7)
O2	0.0642 (10)	0.0364 (8)	0.0347 (9)	0.0084 (8)	−0.0096 (8)	0.0001 (7)
C1	0.0422 (10)	0.0317 (10)	0.0303 (11)	−0.0025 (8)	0.0127 (8)	0.0006 (8)
C2	0.0327 (9)	0.0345 (10)	0.0287 (10)	−0.0030 (7)	0.0109 (8)	−0.0038 (8)
C3	0.0235 (8)	0.0298 (9)	0.0251 (9)	0.0000 (7)	0.0041 (7)	−0.0019 (7)
C4	0.0317 (10)	0.0282 (9)	0.0325 (11)	−0.0004 (7)	0.0028 (8)	−0.0044 (8)
C5	0.0214 (8)	0.0275 (8)	0.0253 (10)	−0.0013 (6)	0.0055 (6)	−0.0015 (7)
C6	0.0375 (10)	0.0303 (9)	0.0261 (10)	−0.0009 (8)	0.0099 (8)	0.0006 (8)
C7	0.0330 (9)	0.0331 (10)	0.0257 (10)	0.0025 (8)	0.0084 (7)	0.0008 (8)
C8	0.0255 (9)	0.0299 (9)	0.0261 (10)	0.0024 (7)	0.0048 (7)	−0.0035 (7)
C9	0.0271 (9)	0.0286 (9)	0.0251 (10)	0.0010 (7)	0.0020 (7)	−0.0001 (7)
C10	0.0230 (7)	0.0274 (8)	0.0261 (10)	−0.0010 (7)	0.0054 (6)	0.0006 (7)
C11	0.0400 (11)	0.0332 (10)	0.0326 (11)	0.0057 (9)	−0.0041 (8)	0.0012 (8)
C12	0.0414 (11)	0.0271 (9)	0.0328 (11)	0.0005 (8)	0.0041 (9)	0.0021 (8)
C13	0.0393 (11)	0.0255 (9)	0.0406 (12)	0.0021 (8)	0.0020 (9)	−0.0017 (9)
C14	0.0347 (10)	0.0319 (11)	0.0397 (12)	0.0048 (8)	0.0034 (9)	−0.0086 (8)
C15	0.0312 (9)	0.0332 (10)	0.0278 (10)	0.0008 (8)	0.0047 (7)	−0.0048 (8)
C16	0.0708 (17)	0.0302 (11)	0.0520 (16)	0.0069 (11)	−0.0024 (13)	0.0029 (11)
C17	0.0303 (10)	0.0403 (11)	0.0425 (13)	0.0010 (8)	0.0126 (8)	−0.0066 (10)
C18	0.0462 (12)	0.0343 (11)	0.0475 (15)	0.0059 (9)	0.0095 (10)	−0.0106 (10)
C19	0.0560 (14)	0.0314 (10)	0.0429 (14)	−0.0048 (10)	0.0130 (11)	−0.0010 (10)
C20	0.0227 (8)	0.0373 (10)	0.0413 (12)	−0.0006 (8)	0.0046 (8)	−0.0054 (9)

### Geometric parameters (Å, °)

**Table:**

O1—C13	1.443 (3)	C10—H10	1.00
O1—H1	0.99 (3)	C11—C12	1.507 (3)
O2—C7	1.215 (3)	C11—H11A	0.99
C1—C10	1.525 (3)	C11—H11B	0.99
C1—C2	1.546 (3)	C12—C13	1.516 (3)
C1—H1A	0.99	C12—H12	1.00
C1—H1B	0.99	C13—C16	1.524 (3)
C2—C3	1.548 (3)	C13—C14	1.525 (4)
C2—H2A	0.99	C14—C15	1.523 (3)
C2—H2B	0.99	C14—H14A	0.99
C3—C4	1.538 (3)	C14—H14B	0.99
C3—C5	1.553 (3)	C15—H15A	0.99
C3—H3	1.00	C15—H15B	0.99
C4—C19	1.525 (3)	C16—H16A	0.98
C4—C18	1.529 (3)	C16—H16B	0.98
C4—H4	1.00	C16—H16C	0.98
C5—C20	1.535 (2)	C17—H17A	0.98
C5—C10	1.538 (3)	C17—H17B	0.98
C5—C6	1.547 (3)	C17—H17C	0.98
C6—C7	1.516 (3)	C18—H18A	0.98
C6—H6A	0.99	C18—H18B	0.98
C6—H6B	0.99	C18—H18C	0.98
C7—C8	1.532 (3)	C19—H19A	0.98
C8—C15	1.539 (3)	C19—H19B	0.98
C8—C9	1.547 (3)	C19—H19C	0.98
C8—C17	1.553 (3)	C20—H20A	0.98
C9—C11	1.514 (3)	C20—H20B	0.98
C9—C10	1.520 (3)	C20—H20C	0.98
C9—C12	1.521 (3)		
			
C13—O1—H1	107.5 (18)	C12—C11—H11A	117.7
C10—C1—C2	104.67 (16)	C9—C11—H11A	117.7
C10—C1—H1A	110.8	C12—C11—H11B	117.7
C2—C1—H1A	110.8	C9—C11—H11B	117.7
C10—C1—H1B	110.8	H11A—C11—H11B	114.8
C2—C1—H1B	110.8	C11—C12—C13	121.3 (2)
H1A—C1—H1B	108.9	C11—C12—C9	60.01 (14)
C1—C2—C3	106.74 (17)	C13—C12—C9	122.7 (2)
C1—C2—H2A	110.4	C11—C12—H12	114.1
C3—C2—H2A	110.4	C13—C12—H12	114.1
C1—C2—H2B	110.4	C9—C12—H12	114.1
C3—C2—H2B	110.4	O1—C13—C12	105.05 (18)
H2A—C2—H2B	108.6	O1—C13—C16	109.21 (19)
C4—C3—C2	112.18 (16)	C12—C13—C16	110.81 (19)
C4—C3—C5	118.79 (15)	O1—C13—C14	109.27 (18)
C2—C3—C5	103.20 (15)	C12—C13—C14	111.03 (18)
C4—C3—H3	107.4	C16—C13—C14	111.26 (19)
C2—C3—H3	107.4	C15—C14—C13	111.42 (17)
C5—C3—H3	107.4	C15—C14—H14A	109.3
C19—C4—C18	109.33 (18)	C13—C14—H14A	109.3
C19—C4—C3	114.03 (18)	C15—C14—H14B	109.3
C18—C4—C3	109.55 (17)	C13—C14—H14B	109.3
C19—C4—H4	107.9	H14A—C14—H14B	108
C18—C4—H4	107.9	C14—C15—C8	111.71 (16)
C3—C4—H4	107.9	C14—C15—H15A	109.3
C20—C5—C10	111.46 (15)	C8—C15—H15A	109.3
C20—C5—C6	112.37 (17)	C14—C15—H15B	109.3
C10—C5—C6	107.48 (15)	C8—C15—H15B	109.3
C20—C5—C3	109.86 (16)	H15A—C15—H15B	107.9
C10—C5—C3	100.68 (14)	C13—C16—H16A	109.5
C6—C5—C3	114.39 (16)	C13—C16—H16B	109.5
C7—C6—C5	114.38 (16)	H16A—C16—H16B	109.5
C7—C6—H6A	108.7	C13—C16—H16C	109.5
C5—C6—H6A	108.7	H16A—C16—H16C	109.5
C7—C6—H6B	108.7	H16B—C16—H16C	109.5
C5—C6—H6B	108.7	C8—C17—H17A	109.5
H6A—C6—H6B	107.6	C8—C17—H17B	109.5
O2—C7—C6	119.85 (18)	H17A—C17—H17B	109.5
O2—C7—C8	121.02 (18)	C8—C17—H17C	109.5
C6—C7—C8	118.94 (17)	H17A—C17—H17C	109.5
C7—C8—C15	110.17 (16)	H17B—C17—H17C	109.5
C7—C8—C9	110.04 (15)	C4—C18—H18A	109.5
C15—C8—C9	110.73 (16)	C4—C18—H18B	109.5
C7—C8—C17	102.90 (17)	H18A—C18—H18B	109.5
C15—C8—C17	108.97 (16)	C4—C18—H18C	109.5
C9—C8—C17	113.75 (17)	H18A—C18—H18C	109.5
C11—C9—C10	120.53 (17)	H18B—C18—H18C	109.5
C11—C9—C12	59.54 (14)	C4—C19—H19A	109.5
C10—C9—C12	116.15 (17)	C4—C19—H19B	109.5
C11—C9—C8	118.43 (17)	H19A—C19—H19B	109.5
C10—C9—C8	113.08 (16)	C4—C19—H19C	109.5
C12—C9—C8	119.15 (17)	H19A—C19—H19C	109.5
C9—C10—C1	120.82 (17)	H19B—C19—H19C	109.5
C9—C10—C5	113.78 (16)	C5—C20—H20A	109.5
C1—C10—C5	104.67 (15)	C5—C20—H20B	109.5
C9—C10—H10	105.4	H20A—C20—H20B	109.5
C1—C10—H10	105.4	C5—C20—H20C	109.5
C5—C10—H10	105.4	H20A—C20—H20C	109.5
C12—C11—C9	60.45 (13)	H20B—C20—H20C	109.5
			
C10—C1—C2—C3	3.7 (2)	C12—C9—C10—C1	−38.2 (2)
C1—C2—C3—C4	152.34 (17)	C8—C9—C10—C1	178.76 (16)
C1—C2—C3—C5	23.3 (2)	C11—C9—C10—C5	−95.5 (2)
C2—C3—C4—C19	−173.09 (18)	C12—C9—C10—C5	−164.01 (16)
C5—C3—C4—C19	−52.7 (2)	C8—C9—C10—C5	53.0 (2)
C2—C3—C4—C18	64.0 (2)	C2—C1—C10—C9	−159.73 (17)
C5—C3—C4—C18	−175.61 (18)	C2—C1—C10—C5	−29.8 (2)
C4—C3—C5—C20	−48.0 (2)	C20—C5—C10—C9	61.6 (2)
C2—C3—C5—C20	76.87 (19)	C6—C5—C10—C9	−62.0 (2)
C4—C3—C5—C10	−165.61 (16)	C3—C5—C10—C9	178.04 (16)
C2—C3—C5—C10	−40.77 (17)	C20—C5—C10—C1	−72.4 (2)
C4—C3—C5—C6	79.5 (2)	C6—C5—C10—C1	164.08 (15)
C2—C3—C5—C6	−155.67 (15)	C3—C5—C10—C1	44.09 (17)
C20—C5—C6—C7	−106.20 (19)	C10—C9—C11—C12	−104.3 (2)
C10—C5—C6—C7	16.8 (2)	C8—C9—C11—C12	108.9 (2)
C3—C5—C6—C7	127.63 (17)	C9—C11—C12—C13	−112.3 (2)
C5—C6—C7—O2	−148.2 (2)	C10—C9—C12—C11	111.6 (2)
C5—C6—C7—C8	36.7 (3)	C8—C9—C12—C11	−107.7 (2)
O2—C7—C8—C15	16.3 (3)	C11—C9—C12—C13	110.0 (2)
C6—C7—C8—C15	−168.73 (18)	C10—C9—C12—C13	−138.4 (2)
O2—C7—C8—C9	138.6 (2)	C8—C9—C12—C13	2.3 (3)
C6—C7—C8—C9	−46.4 (2)	C11—C12—C13—O1	173.55 (19)
O2—C7—C8—C17	−99.8 (2)	C9—C12—C13—O1	101.3 (2)
C6—C7—C8—C17	75.2 (2)	C11—C12—C13—C16	−68.6 (3)
C7—C8—C9—C11	150.51 (18)	C9—C12—C13—C16	−140.9 (2)
C15—C8—C9—C11	−87.4 (2)	C11—C12—C13—C14	55.5 (3)
C17—C8—C9—C11	35.7 (3)	C9—C12—C13—C14	−16.7 (3)
C7—C8—C9—C10	1.4 (2)	O1—C13—C14—C15	−67.2 (2)
C15—C8—C9—C10	123.40 (17)	C12—C13—C14—C15	48.2 (2)
C17—C8—C9—C10	−113.48 (19)	C16—C13—C14—C15	172.11 (18)
C7—C8—C9—C12	−140.47 (18)	C13—C14—C15—C8	−68.4 (2)
C15—C8—C9—C12	−18.4 (2)	C7—C8—C15—C14	172.31 (18)
C17—C8—C9—C12	104.7 (2)	C9—C8—C15—C14	50.3 (2)
C11—C9—C10—C1	30.3 (3)	C17—C8—C15—C14	−75.5 (2)

### Hydrogen-bond geometry (Å, °)

**Table:**

*D*—H···*A*	*D*—H	H···*A*	*D*···*A*	*D*—H···*A*
O1—H1···O2^i^	0.99 (3)	1.93 (3)	2.916 (2)	172 (3)

Symmetry codes: (i) −*x*+2, *y*+1/2, −*z*+1.
